# Large-scale cortico-cerebellar computations for horizontal and vertical vergence in humans

**DOI:** 10.1038/s41598-022-15780-9

**Published:** 2022-07-08

**Authors:** Hiroyuki Mitsudo, Naruhito Hironaga, Katsuya Ogata, Shozo Tobimatsu

**Affiliations:** 1 Division of Psychology, Department of Human Sciences, Faculty of Human-Environment Studies, Kyushu University, 744 Moto-oka, Nishi-ku, Fukuoka, 819-0395 Japan; 2 Department of Clinical Neurophysiology, Graduate School of Medical Sciences, Kyushu University, 3-1-1 Maidashi, Higashi-Ku, Fukuoka, Fukuoka 812-8582 Japan; 3 Department of Pharmaceutical Sciences, School of Pharmacy at Fukuoka, International University of Health and Welfare, Enokizu 137-1, Okawa, 831-8501 Japan; 4 Department of Orthoptics, Faculty of Medicine, Fukuoka International University of Health and Welfare, 3-6-40 Momochihama, Sawara-Ku, Fukuoka, Fukuoka 814-0001 Japan

**Keywords:** Visual system, Sensory processing, Oculomotor system

## Abstract

Horizontal and vertical vergence eye movements play a central role in binocular coordination. Neurophysiological studies suggest that cortical and subcortical regions in animals and humans are involved in horizontal vergence. However, little is known about the extent to which the neural mechanism underlying vertical vergence overlaps with that of horizontal vergence. In this study, to explore neural computation for horizontal and vertical vergence, we simultaneously recorded electrooculography (EOG) and whole-head magnetoencephalography (MEG) while presenting large-field stereograms for 29 healthy human adults. The stereograms were designed to produce vergence responses by manipulating horizontal and vertical binocular disparities. A model-based approach was used to assess neural sensitivity to horizontal and vertical disparities via MEG source estimation and the theta-band (4 Hz) coherence between brain activity and EOG vergence velocity. We found similar time-locked neural responses to horizontal and vertical disparity in cortical and cerebellar areas at around 100–250 ms after stimulus onset. In contrast, the low-frequency oscillatory neural activity associated with the execution of vertical vergence differed from that of horizontal vergence. These findings indicate that horizontal and vertical vergence involve partially shared but distinct computations in large-scale cortico-cerebellar networks.

## Introduction

Binocular coordination is mandatory for stereoscopic vision. One type of binocular coordination is horizontal vergence, which is a disconjugate eye movement in the lateral direction. The two eyes converge when fixating on a near object relative to the head, and diverge when fixating on a far object. Horizontal vergence is typically induced by the lateral displacement of visual elements between the two eyes’ retinae, called horizontal binocular disparity^1–3^. Many visual areas (V1-V4, and MT+) show sensitivity to binocular disparities^4–11^. Furthermore, several neurophysiological studies indicate that horizontal vergence is influenced by cortical, cerebellar, and brainstem regions. Single-cell recording studies have suggested that neurons in the frontal eye field^12^, the cerebellum^13,14^, and the midbrain^15,16^ of monkeys are likely to be involved in generating horizontal vergence signals. This view is generally consistent with human data using electroencephalography (EEG) recordings before vergence^17^ and transcranial magnetic stimulation (TMS) to the cerebellum^18^.

Recent behavioral studies have accumulated evidence that vergence depends on stimulus. Large-field random-dot stereograms, instead of the sparse stimuli used in earlier neurophysiological studies (e.g., a single point light source), could produce fast and involuntary disparity-induced vergence. The fast vergence produced by large-field stereograms occurred vertically as well as horizontally in an almost linear response to disparity magnitudes of less than one degree of visual angle^ (dva) 19–21^. Interestingly, there are some important differences between horizontal and vertical vergence. For example, in contrast to horizontal vergence, human subjects cannot control vertical vergence voluntarily^22^. In addition, subjects are normally unaware of both the occurrence of vertical vergence^2^ and the presence of vertical disparity^23^. The range of vertical vergence is smaller than that of horizontal vergence^2^. To date, however, little is known about the extent to which the neural mechanism underlying vertical vergence overlaps with that of horizontal vergence. Our goal was to examine whether disparity-induced vertical vergence is largely an automatic process for binocular coordination and involves somewhat different mechanisms compared with those for horizontal vergence.

**Figure 1:**
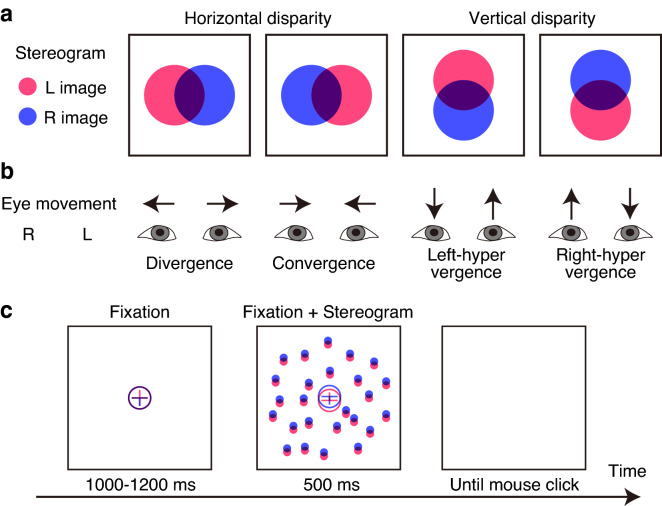
Schematic illustration of the visual stimuli, eye movement, and trial sequence. (**a**) Four stimulus conditions. Red and blue disks indicate the elements presented to the left and right eyes, respectively. (**b**) The corresponding four types of disparity-induced vergence. (**c**) An example of the trial sequence (the right-hyper vertical disparity condition). After a presentation of the fixation pattern, a random-dot stereogram was presented for 500 ms. The subjects’ primary task was to keep their eyes on the fixation pattern.

In this study, we explored neural computations for disparity-induced horizontal and vertical vergence in humans by recording whole-head magnetoencephalography (MEG) responses from healthy subjects while presenting large-field stereograms. In addition to the MEG, we measured electrooculography (EOG) in order to analyze binocular eye movements. To fully present binocular cues for vergence, we used static random-dot stereograms. The stereograms were designed to produce disparity-induced vergence responses in the horizontal or vertical direction. Figure 1 shows an overview of the visual stimuli and the experimental procedure used in this study. The first step of our analysis was to identify the spatiotemporal profile of MEG source activity in response to horizontal or vertical disparities. We included cerebellar regions in our analyses because (1) single-unit recording studies indicated a contribution of the cerebellum in vergence control^14,24^, and (2) MEG can be used for investigating the functional role of the cerebellum in combination with source or network analysis^25^. Subsequently, we searched the brain regions involved in the execution of vergence, by calculating “brain-ocular” coherence with a beamforming technique in the frequency domain (the dynamic imaging of coherent sources, DICS^26^).

To separate brain responses related to vergence from those to disparity, neural sensitivity was assessed using a model-based approach, which analyzes correlations between MEG responses and stimulus parameters^27^. Using this approach, we controlled for neural and ocular response components unrelated to disparity-induced vergence. Based on the assumption that a larger magnitude of disparity elicits a greater neural activity for vergence, the MEG activity was regressed to disparity magnitude. Then, the brain-ocular coherence was regressed to linear disparity in order to isolate the neural signals for vergence execution. Furthermore, since coupling between behavioral performance and neural activity was reported at the single-trial level^28^, we also analyzed trial-by-trial coherence changes associated with the degree to which vergence occurred in response to disparity.

## Results

### Slant discrimination performance

**Figure 2:**
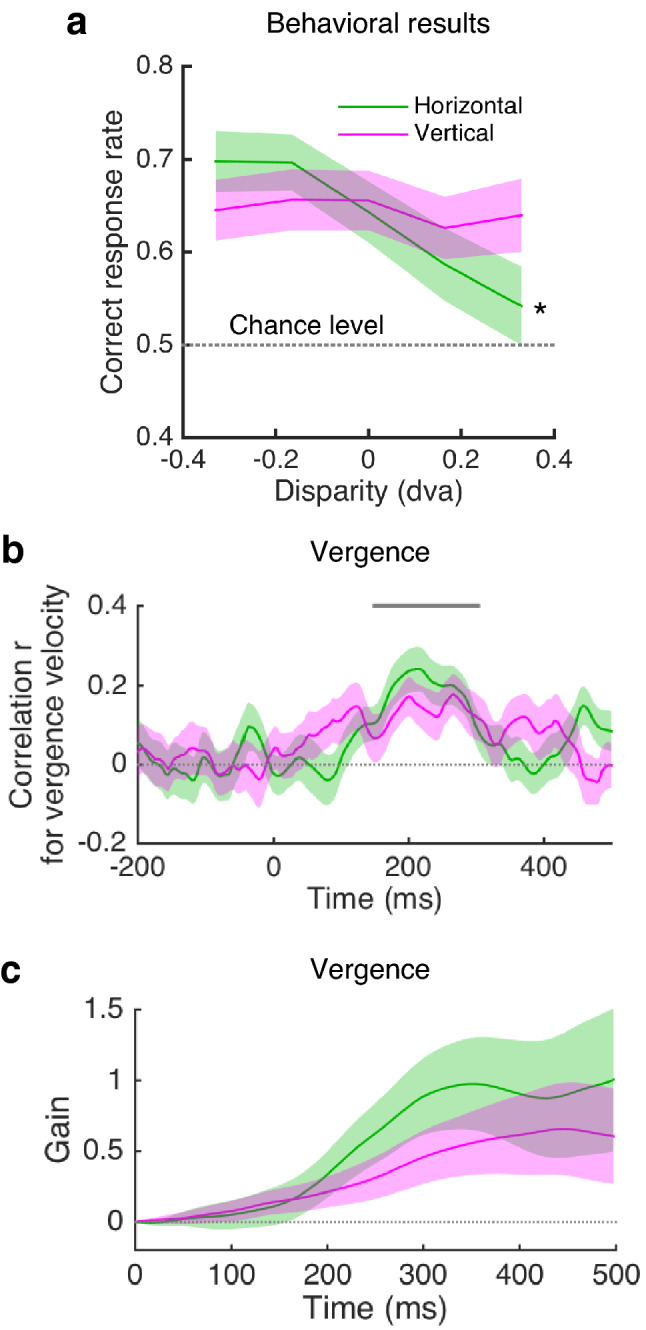
Behavioral and eye movement results (*n* = 29). (**a**) Behavioral performance for slant discrimination in the horizontal and vertical disparity conditions. (**b**) Mean correlation coefficients for vergence velocity averaged over the 29 subjects calculated as a function of time. The horizontal gray bar indicates the interval in which correlation was significantly greater than 0 in the horizontal or vertical disparity condition (150–314 ms; *p*s < 0.05, corrected). (**c**) Mean gain of vergence eye movement relative to stimulus disparity calculated as a function of time. A gain value of 1 indicates the eye position where the two eyes counteract the stimulus disparity perfectly. Color-shaded areas represent the standard errors of the mean (SEMs).

To facilitate binocular fusion, we asked subjects to perform a stereoscopic slant discrimination task during the MEG and EOG measurements. Figure 2 depicts the results of behavioral slant discrimination performance and vergence eve movements (*n* = 29). As seen in Fig. 2a, the mean correct response rates for slant discrimination were affected by horizontal disparity (within-subjects analysis of variance, *F*(4,112) = 15.7, *p* < 0.0001) and not by vertical disparity (*F*(4,112) = 0.76, *p* = 0.55). In the horizontal condition, the correct response rates were significantly higher for crossed disparities than for uncrossed disparities with respect to the fixation pattern (*p* values < 0.0001, multiple comparisons). In sum, slant discrimination performance was well above the chance level (0.5) in both the horizontal and vertical conditions and was affected by horizontal disparity. These data suggest that binocular fusion is maintained during stimulus presentation in almost all conditions.

### Vergence eye movements

To examine whether disparity-induced vergence occurred, we performed a linear regression of vergence velocity against stimulus disparity separately for the horizontal and vertical conditions. Figure 2b shows that disparity-induced vergence occurred in response to the presentation of horizontal and vertical disparities (150–302 ms relative to stimulus onset for horizontal, *p* = 0.002; 232–314 ms for vertical, *p* = 0.033, corrected). The correlation coefficients did not significantly differ between the two disparity conditions for 0–500 ms (two-tailed tests: *p* = 0.67, corrected). Table 1 summarizes the mean and peak vergence velocities averaged over the 29 subjects. The regression coefficients were used to calculate the change of binocular eye position so as to estimate the gain of vergence separately for the horizontal and vertical directions (Fig. 2c). The mean gain values increased as a function of time and approached 1.0 especially in the horizontal condition. The gain values at any time point within 0–500 ms did not differ significantly between the two disparity conditions (two-tailed *t* tests, |*t*(28)| values < 1.42, *p* values > 0.16). Therefore, vergence responses were confirmed at the group level and proportional to stimulus disparity, irrespective of disparity directions.

**Table 1:** Summary of vergence velocity (*n* = 29).

Disparity (dva)	− 0.33	− 0.16	0	0.16	0.33
**Horizontal**					
Mean (deg/s)	− 1.81	0.43	1.18	1.73	1.75
Peak (deg/s)	− 10.81	− 8.99		7.97	8.47
**Vertical**					
Mean (deg/s)	− 0.59	− 0.21	− 0.45	0.83	1.11
Peak (deg/s)	− 3.14	− 2.24		3.36	3.23

Mean velocities were the averaged values over the period during which significant correlations were found between disparity and vergence velocity in each of the horizontal and vertical conditions. Peak velocities in the negative and positive disparity conditions were the group mean of the minimal and maximal values within the significant period, respectively. The averaged *SE* over the disparity conditions was 1.42 and 1.49 deg/s for mean and peak velocities, respectively.

### Time-locked source analysis

**Figure 3:**
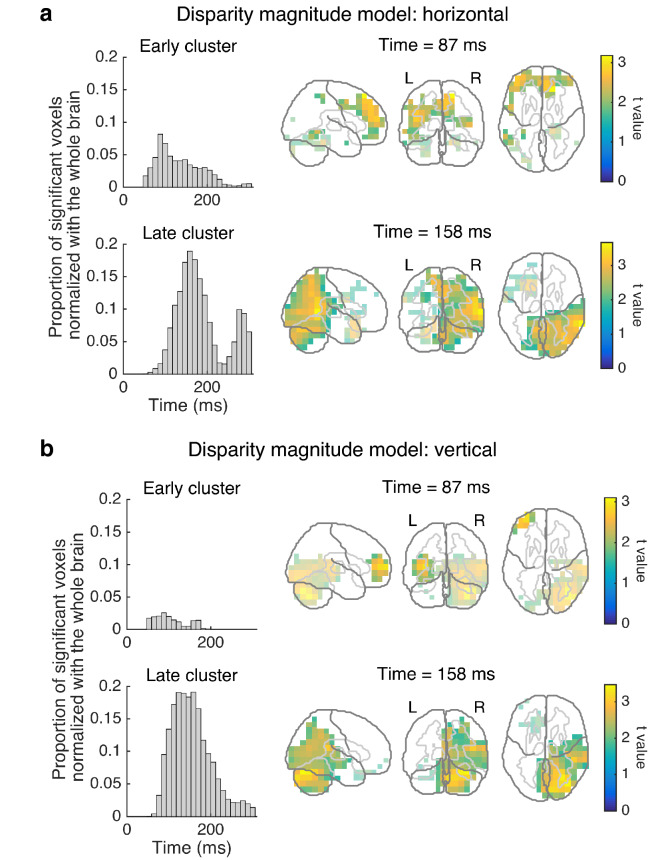
Large-scale neural sensitivity to disparity magnitude. (**a**) The horizontal condition. (**b**) The vertical condition. In each of (**a**) and (**b**), the left two histograms show the early- and late-peak subclusters of the significant voxels showing a positive sensitivity to disparity magnitude (*p* < 0.05, corrected). The right two panels show snapshots of the corresponding cluster in the frontal (top), temporal, occipital, and cerebellar (bottom) areas. The most significant voxels are mapped onto three planes. Light-colored voxels represent the other subcluster (i.e., the late-peak subcluster in the top-right panel and vice versa).

The eye-movement analysis suggested that vergence occurred for ~ 150–300 ms after stimulus onset. To identify time-locked neural activity that preceded or triggered vergence, we calculated source-localized responses averaged in each of the 10 disparity conditions. Since a larger magnitude of disparity was expected to elicit a greater neural activity for vergence irrespective of the disparity sign, we regressed source-localized activity to the disparity magnitude (i.e., the absolute value of disparity). In the horizontal condition, for 50–300 ms relative to stimulus onset, we found a significant cluster where activity over several areas was positively correlated with disparity magnitude (*p* = 0.036, corrected). To interpret the timing and areas of activity, we applied the *k*-means clustering algorithm (*k* = 2) to the temporal array of the significant voxels. As seen in Fig. 3a, one subcluster was located mainly in the left middle frontal area with a relatively early peak of 87 ms, and the other subcluster was located in the right middle temporal and cerebellar areas with a later peak of 158 ms.

Next, we applied the same analysis pipeline to the data for the vertical condition. Again, we found a significant cluster where activity was positively correlated with disparity magnitude (*p* = 0.044, corrected). The subsequent *k*-means clustering analysis (*k* = 2, Fig. 3b) revealed that, as in the horizontal condition, an early-peak subcluster was located largely in the left middle frontal area (peak = 87 ms), and the other late-peak subcluster was located in the right middle temporal, lingual, and cerebellar areas (peak = 158 ms).

### Brain-ocular coherence

**Figure 4:**
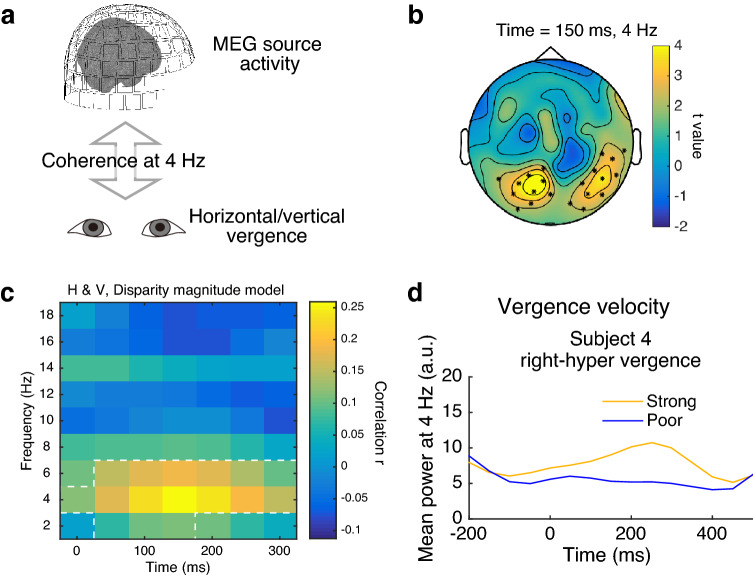
Brain-ocular coherence analysis. (**a**) Schematic illustration of brain-ocular coherence. (**b**) Induced power change associated with disparity magnitude found in occipital sensors. Asterisks indicate significant sensors revealed by a cluster-based permutation test. (**c**) Mean correlations calculated for the asterisked sensors in (b). The white dotted region indicates the significant combination of time and frequency. (**d**) Sample of vergence velocity power for the stimulus-vergence interaction model.

The time-locked source analysis indicated two common components of the neural activity related to disparity-induced vergence in both the horizontal and vertical directions. Since the early subclusters peaked before vergence occurred (~ 100 ms), the first component may reflect the encoding of vergence direction and size before vergence execution. Since the late subclusters peaked around at the beginning of vergence (~ 150 ms, Fig. 2b) and lasted until the end of vergence (~ 300 ms), the second component may reflect the execution of a vergence command for ocular muscle movements. To identify the activity involved in the execution of a vergence command, we analyzed MEG and vergence data together. Figure 4a schematically shows the DICS approach^26^ used to seek brain regions coherent with EOG vergence velocity signals. Using sensor-level MEG data, we first explored the time–frequency range involved in generating signals for the execution of disparity-induced vergence. Since Fig. 3 suggests a large overlap of source-localized responses between the two disparity conditions, the induced power responses were regressed to the magnitude of disparity in both the horizontal and vertical conditions. As found in Fig. 4b,c, the induced power was positively correlated with disparity magnitude in the occipital sensors at 2–6 Hz for 0–300 ms (peak = 4 Hz; two-tailed tests: *p* = 0.014, corrected). Therefore, we calculated coherence at 4 Hz between MEG source activity and EOG vergence velocity.

**Figure 5:**
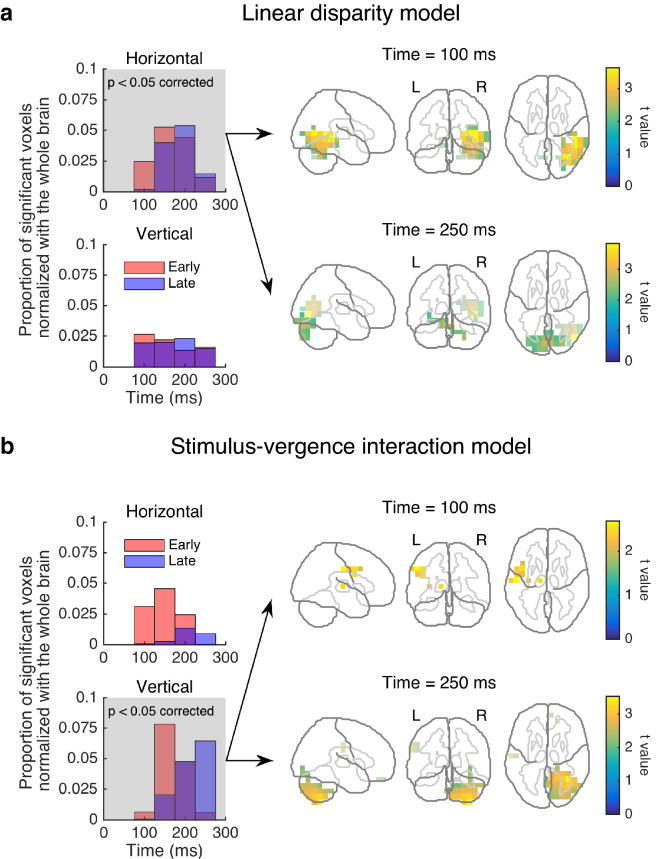
Oscillatory activities related to the execution of disparity-induced vergence. (**a**) Linear disparity model explains the 4-Hz coherence in the right middle temporal and cerebellar areas for the horizontal condition. (**b**) Stimulus-vergence interaction model explains the 4-Hz coherence in the left postcentral and right cerebellar areas for the vertical condition. Each panel in the left column shows the two subclusters of the voxels revealed by each model applied to the horizontal or vertical condition. Shaded panels show the significant clusters (*p* < 0.05, corrected). Right panels show snapshots of the significant clusters revealed by each model. The most significant voxels are mapped onto three planes. Light-colored voxels represent the other subcluster (i.e., the late-peak subcluster in the top-right panel and vice versa).

Figure 5a shows a summary of the results obtained with the linear disparity model. The 4-Hz brain-ocular coherence was higher for convergence than for divergence at 100–250 ms (two-tailed tests: *p* = 0.02, corrected). The *k*-means clustering analysis (*k* = 2) suggests that one subcluster was located mostly in the right middle temporal area (the top-right panel of Fig. 5a), while the other subcluster was located mostly in the cerebellum at similar latencies (the bottom-right panel of Fig. 5a). The bottom-left panel of Fig. 5a shows that the linear disparity model was not significant for vertical disparity (i.e., there was no difference in coherence between left-hyper and right-hyper vertical vergence responses; two-tailed tests: *p* = 0.12, corrected). These results indicate that disparity processing plays a prominent role in the execution of horizontal vergence through theta-band oscillations in right middle temporal and cerebellar areas.

To explore the neural signature of a vergence command further, we examined another model in which the brain-ocular coherence reflects an interaction between stimulus disparity and motor vergence responses. In the stimulus-vergence interaction model, we used trial-by-trial fluctuations in terms of the occurrence of disparity-induced vergence. We first categorized trials according to the degree to which vergence occurred in response to disparity (Fig. 2b). Then, we compared the 4-Hz brain-ocular coherence in trials where disparity-induced vergence occurred strongly with that in trials where disparity-induced vergence occurred poorly. Figure 4d shows an example of the induced power of vergence velocity at 4 Hz for one subject. In the horizontal condition (the top-left panel of Fig. 5b), no significant cluster was found where the 4-Hz brain-ocular coherence changed according to the occurrence of vergence (*p* = 0.32, corrected). However, in the vertical condition (the bottom-left and right panels of Fig. 5b), 4-Hz coherence increased in several areas including the left postcentral area and the right cerebellum for 100–250 ms when vergence occurred strongly in response to vertical disparity (two-tailed tests: *p* = 0.036, corrected). The *k*-means clustering analysis revealed that the late-peak subcluster was mainly the right cerebellum. Since the temporal profile of the late-peak subcluster is consistent with the timing of vertical vergence (Fig. 2b), these results indicate that cerebellar activity plays a role in the execution of disparity-induced vergence in the vertical direction.

## Discussion

To elucidate the neural mechanisms underlying horizontal and vertical vergence, we applied model-based analyses to MEG responses and EOG vergence signals. Our EOG analysis confirmed vergence responses to horizontal and vertical disparities at ~ 150–300 ms after stimulus onset. In both the horizontal and vertical conditions, neural sensitivity to disparity magnitude was found mainly in the left middle frontal area at ~ 100 ms and in the right middle temporal and cerebellar areas at ~ 150–300 ms. Linear horizontal disparity, not vertical disparity, predicted the 4-Hz coherence between vergence velocity and the activity in right middle temporal and cerebellar areas at ~ 200 ms. For stimuli with non-zero vertical disparities, the occurrence of disparity-induced vergence in single trials was correlated with the 4-Hz coherence between vergence velocity and the activity in the right cerebellum at ~ 250 ms. Overall, these data suggest that horizontal and vertical vergence involve partially shared but different neural computations across cortical and subcortical areas.

The present time-locked MEG responses showed similar patterns for horizontal and vertical disparities before vergence occurred (at ~ 100–150 ms after stimulus onset). These results are somewhat inconsistent with the previous studies reporting stronger EEG responses for horizontal than for vertical in adult participants^29,30^. Note that, whereas the current study used the vertical disparity that did not impair depth perception, the previous studies used the vertical disparity that produced an ambiguous depth perception. Given the stimulus differences, weaker neural responses to vertical disparity reported in the previous studies are likely to result from the reduced depth perception. This interpretation is in line with a recent MEG study that used the vertical size disparity designed to produce a stereoscopic slant^31^. The present results therefore provide neuromagnetic evidence for the idea suggested by behavioral studies^32^, in which the global stimulus configuration plays a role in computing binocular disparity not only along horizontal but also along vertical orientations.

The time-locked source analysis also revealed the sensitivity to disparity magnitude in the right middle temporal area (i.e., at ~ 100–150 ms relative to stimulus onset). These results are generally in line with a functional magnetic resonance imaging (fMRI) study in which horizontal disparity magnitude is encoded in dorsal visual areas^33^. However, we found that, for sensitivity to vertical disparity (Fig. 3b), the peak location within the cerebral cortex was not the middle temporal area, but the lingual gyrus. Since this area overlaps with the ventral visual pathway, our results support the recent view that disparity processing involves cortical interactions among multiple visual areas^34^.

In addition to disparity magnitude, neural sensitivity to disparity sign is required to execute disparity-induced vergence^19^. This pattern of sensitivity was found on the 4-Hz brain-ocular coherence for linear horizontal disparity with a time window of ~ 200 ms (Fig. 5a), in which disparity-induced vergence was found (Fig. 2b). That cluster peaked in the right middle temporal area, which partially overlapped with those revealed by the disparity magnitude model described above. These results are in line with a macaque study^35^ showing that the middle temporal and medial superior temporal areas are involved in the execution of horizontal vergence (but not with a human fMRI study^36^).

The time-locked source analysis revealed the activation of mainly the left middle frontal area at ~ 100 ms in both the horizontal and vertical conditions. This activity preceded the vergence eye movements, and thus may be involved in the coding of vergence size. This interpretation is consistent with other studies indicating that the frontal eye field is involved in generating signals for eye movements such as vergence as well as saccades^37^. Furthermore, in both the horizontal and vertical conditions, the two different source reconstruction approaches consistently suggested an involvement of the right cerebellum at 200–250 ms (Figs. 3, 5). These results extend earlier studies using human TMS^18^ and macaque single-cell recordings^14^, by providing neuromagnetic evidence for the contribution of the cerebellum to vergence eye movements.

Whereas the results overlap partially between horizontal and vertical vergence, some differences exist. The brain-ocular coherence analysis suggested that neural sensitivity to linear (i.e., signed) disparity was found in the horizontal, not in the vertical condition. As described in Fig. 5a, the sensitivity to signed horizontal disparity spanned right middle temporal and cerebellar areas at ~ 200 ms relative to stimulus onset. Since this timing overlaps with the timing of disparity-induced vergence (Fig. 2b), this sensitivity probably reflects neural commands for the execution of vergence. We interpret the brain-ocular coherence at 4 Hz as reflecting theta-band oscillations across long-range connections between middle temporal, cerebellar, and other areas. This interpretation is generally in line with a known feature of theta-band oscillations^38–40^. With the definition of coherence by Gross et al*.*^26^, one can also regard the brain-ocular coherence as reflecting the neuronal communications between the eyes and specific brain areas. Considering the behavioral results demonstrating fast vergence responses to disparity at a shorter visual presentation^19^ (~ 70 ms), we tentatively suggest that horizontal vergence involves feedforward processing from the left middle frontal area to the right middle temporal area and the cerebellum, and thus contributes to rapid object recognition in 3-D space. Note that our static stereograms can be processed via two separate mechanisms, which encode disparity signals and monocularly trackable features independently^41^. Since we observed transient vergence responses at ~ 150–300 ms after stimulus onset, we speculate that the neural responses found here are mainly triggered by the onset of disparity cues rather than by the continuous processing of monocularly trackable features. To test this idea experimentally, it will be helpful to use dynamic stereograms that can induce vergence in future neuroimaging studies.

The behavioral slant discrimination performance was asymmetrical between convergence and divergence (Fig. 2a). These results are consistent with a known asymmetry in disparity and vergence processing, where visual search performance was better for crossed disparity than for uncrossed disparity^42^ (i.e., near > far), and event-related potential amplitudes were higher for convergence than for divergence^43^. The present neuromagnetic results provide further evidence for this asymmetry, in that the 4-Hz brain-ocular coherence was higher for convergence than for divergence.

Only in trials in which vergence occurred so as to counteract signed vertical disparities, the 4-Hz brain-ocular coherence increased mainly in the right cerebellum at ~ 250 ms (Fig. 5b). This result was obtained using the stimulus-vergence interaction model applied to the coherence data in the vertical, not in the horizontal condition. These coherence results are not explained in terms of vergence velocity because the strong-poor differences in vergence velocity were not significantly greater for vertical than for horizontal (see “Methods”). Notably, as seen in the top-right panel of Fig. 5b, at a shorter latency (~ 100 ms), we also found an increase in coherence around the left postcentral area. A recent study suggests that a connection between the postcentral and visual areas is mediated by proprioceptive eye position signals^44^. Therefore, we speculate that visuo-proprioceptive-motor interactions may contribute to automatic correction for ocular misalignments through vertical vergence.

What underlies the anisotropic processing for horizontal and vertical vergence? We propose that a relatively automatic control of vertical vergence is beneficial for binocular stereo vision. According to psychophysical studies, the visual system primarily uses a horizontally elongated fusional range of stereoscopic matching^45–47^. Sprague et al*.*^34^ pointed out that, across a variety of eccentric gaze positions, naturally occurring vertical disparities are smaller than horizontal disparities. Therefore, the visual system may have circuits that efficiently execute vertical vergence by restricting the possible matching range of vertical disparity. In this scenario, while correcting vertical ocular misalignments automatically, the visual system can focus on horizontal disparity processing in combination with horizontal vergence so as to represent a detailed 3D structure of the visual scene.

This study focused on a relatively small range of vergence responses. Since we did not conduct formal heterophoria tests, some subjects might show abnormal vergence responses particularly to large-disparity stimuli as found in Kapoula et al*.*^48^. By measuring heterophoria in future neuroimaging studies, we will gain a better understanding of the neural mechanisms underlying vergence disorders.

In relatively unrestricted viewing, saccadic and vergence eye movements can occur simultaneously^49^. Whereas our EOG analysis revealed a statistically significant vergence component of binocular eye movements at the group level, we could not analyze potential small saccades in detail. In addition, single-unit recording studies suggest that the midbrain plays a role in executing disjunctive saccades with a vergence component^15^. The present study did not address the involvement of the midbrain because the current MEG source localization techniques become inaccurate for deeper brain areas such as the midbrain. Single-unit recording studies also reported that neurons in the midbrain and cerebellum showed selectivity to vergence angle as well as vergence velocity^14,50^. Therefore, it will be worthwhile to examine the relation between the whole brain activity and vergence angle. Further behavioral, neuroimaging, and computational studies will be necessary to describe a large and detailed picture of binocular coordination.

To summarize, we found similar time-locked neural responses to horizontal and vertical disparity in cortical and cerebellar areas at around 100–250 ms after stimulus onset. In contrast, the low-frequency oscillatory neural activity associated with the execution of vertical vergence differed from that of horizontal vergence. These findings demonstrate partially shared but distinct computations to control horizontal and vertical vergence in binocular coordination.

## Methods

### Subjects

Thirty participants took part in the experiment. One was excluded because of excessive EOG noise during the experiment. Thus, we analyzed data from the remaining 29 participants (age range, 20–29 years; 16 males; 25 right-handed). All subjects had normal or corrected-to-normal (by contact lenses) visual acuity and reported no sensorimotor deficit. None were stereo-blind according to the stereo fly test (Stereo Optical Co. Inc., Chicago, IL, USA), because the individuals’ stereo disparity thresholds were equal or below 140 arc sec of visual angle, which was not the greatest level of the stereo test. The mean stereo threshold was 45 (*SD* = 19) arc sec of visual angle. Since the expected amplitude of vergence was small in this study (≤ 0.33 dva) and was within a typical range of binocular fusion^51^, we expected that small disparity-induced vergence responses would be found in stereo-normal subjects. Therefore, we did not conduct a formal heterophoria test and included subjects who passed the stereo test in order to maximize the sample size (but see also van Rijn et al*.*^52^). The experimental protocol was approved by the ethics committee of the Kyushu University Hospital (No. 20192016). Written informed consent was obtained from all participants. The experiment was carried out in accordance with the latest version of the Declaration of Helsinki.

### Stimuli and procedure

Stereograms were presented on a rear-projection screen using a Digital Light Processing projector (1024 × 768 pixels; H5360BD, Acer, New Taipei City, Taiwan) at 60 Hz. The presentation was controlled by a computer (Macbook Air, Apple Inc., Cupertino, CA, USA). One pixel subtended 0.055 dva. The stereograms consisted of ~ 750 dots presented within a circular region with a diameter of 28 dva. No dot was presented at a central region (diameter, 6.6 dva) of the image. Figure 1a schematically shows binocular disparities created by entirely displacing the position of all elements presented to each eye in the opposite directions to each other. The resulting disparities were − 0.33, − 0.16, 0.00, 0.16, and 0.33 dva. These stereograms were expected to produce the vergence eye movements shown in Fig. 1b. The orientation of displacement was horizontal or vertical. In this paper, a positive value corresponds to an uncrossed disparity (i.e., expected to produce a divergence eye movement) in the horizontal condition and a left-hyper disparity in the vertical condition. In addition to the overall horizontal or vertical disparity, the horizontal size ratio (HSR, the proportion of the width of the left eye’s pattern to that of the right eye’s pattern) was introduced into the whole stimulus so as to produce an overall slant about a vertical axis. HSR values were 1.025 and 1/1.025, corresponding to right-near and left-near slants, respectively. Each dot was drawn in 6 × 6 pixels (before blur) and presented by the antialiasing method. The dots were blurred with a Gaussian kernel (radius, 3.6 pixels). A fixation pattern consisted of a cross surrounded by a circle and subtended 2.4 × 2.4 dva. The vertical line of the fixation was nonius lines. To present anaglyph stereograms, the red and blue images were viewed through spectacles with red and blue filters in front of the left and right eyes, respectively. The mean background luminance was 0.14 cd/m^2^, and the maximal luminance averaged across the two eyes was 7.0 cd/m^2^ (through red-blue spectacles). The crosstalk between the two eyes was 4% on average and small enough to produce the desired disparity.

Figure 1c shows the time course of a trial. The subjects viewed the screen in the upright position of the MEG dewar at a distance of 112 cm in a magnetically shielded room. Since the room was dark apart from the stimulus, objects in the subject’s periphery were almost invisible. At the beginning of each trial the fixation with zero disparity was presented for 1–1.2 s and followed by a presentation of the random-dot stereogram for 500 ms. The fixation remained on the screen until the stereogram disappeared. The disparity of the fixation was kept identical to that of the stereogram. During the stimulus presentation, the subjects were asked to gaze at the fixation pattern and to suppress eye blinks as much as they could. After the stimulus disappeared, the subjects were asked to report which side of the random-dot pattern appeared in front of the fixation with a two-alternative forced-choice procedure. The subjects reported by clicking the left or right button with the index or middle finger of their right hand, respectively. No speedy discrimination was required, and a click triggered the next trial. Each MEG run consisted of 40 trials tested in a randomized order (2 disparity directions × 2 slant orientations × 5 disparities × 2 repetitions). Approximately four practice runs were performed with feedback for incorrect responses without MEG measurement. The MEG session consisted of 11 runs, and the first run served as practice. Therefore, we analyzed the remaining 10 runs.

### EOG measurement and analysis

**Figure 6:**
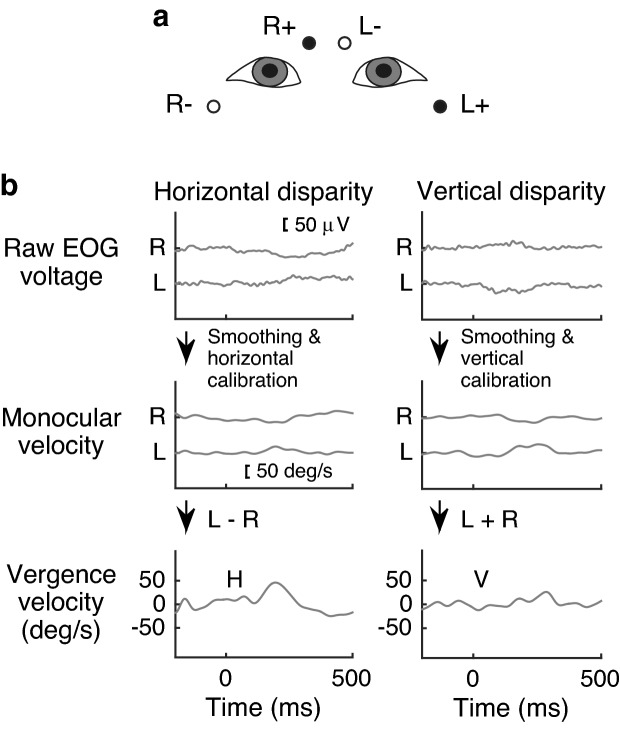
Calculation of vergence eye movements in a trial. (**a**) Approximate position of bipolar EOG electrodes for each eye. (**b**) Sequence of EOG data processing. Sample data are shown for a single trial from one subject separately for each disparity condition (left or right column). Raw voltage data (upper row) were transformed into velocity data (middle row). Vergence velocity was calculated by combining the two eyes’ data (bottom row).

Our EOG measurement had two goals: to detect unwanted large eye movements for artifact rejection^53^ and to measure disparity-induced vergence. To analyze the time course of vergence eye movements, a bipolar EOG was recorded for each eye throughout the MEG runs. Because single-unit recording studies reported that neurons in several areas showed sensitivity to vergence velocity ^12,14,16^, our EOG analysis aimed at computing noise-free vergence velocity from voltage data. Figure 6 summarizes our general procedure to obtain vergence data for each trial. The analysis generally followed the procedure described in Spekreijse and Riemslag^54^. Figure 6a shows the EOG electrode arrangement, where one electrode was placed at a nasal and upper position relative to one eye, and the other electrode at a temporal and lower position relative to the same eye. A bipolar EOG was attached around each eye, so as to estimate horizontal or vertical vergence. The top row of Fig. 6b shows samples of EOG voltage signals. To obtain velocity data for each eye, after being down-sampled to 256 Hz, differentiated voltage signals were calculated for every time point by subtracting the data from the preceding time point in combination with a 9-point calibration procedure (the middle row of Fig. 6b). To reduce high-frequency EOG noises while keeping low-frequency vergence components (in comparison with saccades^55^), the signals were smoothed by calculating the moving-average over 25 time points and then low-pass filtered at 18 Hz. The purpose of the time-averaging procedure was to perform group-level permutation tests for EOG data (see the “Statistical analysis” section) while tolerating some individual differences in the timing of transient vergence responses. Due to excessive noise in signals obtained from two subjects, a moving-average window of 36 time points was used for one subject, and the second-order polynomial component was removed for the other subject. The bottom row of Fig. 6b shows samples of a vergence curve in a trial. Since one-directional (i.e., horizontal or vertical) vergence was assumed to occur with our experimental procedure (i.e., saccades or oblique vergence were not expected), horizontal and vertical vergence velocities were calculated by subtracting and adding the two eyes’ data separately for each of the horizontal and vertical disparity conditions, respectively.

Slow ocular drift components in each trial were removed by subtracting the vergence velocity averaged over a baseline period of − 200 ms to 0 ms relative to stimulus onset from the velocity profiles. To examine ocular sensitivity to disparity and reduce trial-by-trial fluctuations, vergence data were averaged across trials and fitted to linear disparity. The occurrence of horizontal and vertical vergence was examined at the group level by permutation tests similar to those used for MEG data (see the “Statistical analysis” section).

### MEG measurement and analysis

The core parts of MEG measurement settings followed our previous MEG study^31^. In short, data were acquired using a 306-channel MEG system (Neuromag, Elekta, Helsinki, Finland; consisting of 204 planar gradiometers and 102 magnetometers). MEG and EOG signals were continuously recorded at a sampling rate of 1000 Hz with a bandpass filter of 0.1–330 Hz. Four head position indicator (HPI) coils were attached to the subject’s head. The positions of the four HPI coils and the subject’s nasion, left and right pre-auricular points, and head shape were measured with a 3-D digitizer (FASTRAK, Polhemus, VT, USA). The mean lag of visual stimulus onset with respect to the trigger onset was 26 ms and was corrected offline.

To explore brain regions and neural oscillations involved in disparity-induced vergence, we processed MEG data using time-locked source localization, time–frequency decomposition, and coherence analyses. All MEG data were preprocessed using Maxfilter and Maxmove^56,57^ in order to eliminate noise outside the brain and to spatially transform data into the default head position in the MEG dewar. The data analysis generally followed the guidelines proposed by Gross et al*.*^58^ and was performed with the Fieldtrip toolbox^59^ on MATLAB (version R2015, Mathworks, Natick, MA, USA). Data from trials with excessive MEG or EOG noise (e.g., eye blinks and saccades) were removed semi-automatically using visual inspections. Independent component analysis^60^ was used to find and remove artifacts of eye blinks, saccades, and cardiac responses. MEG sensor data were also down-sampled to 256 Hz. In the time-locked source localization and coherence analyses, we used all the magnetometers and gradiometers to improve localization accuracy^61,62^. In the time–frequency analysis, we used all the magnetometers to detect neural signals from the deeper locations in the brain^63^.

### Time-locked source localization analysis

To localize the neural source of event-related electromagnetic responses, we used minimum-norm estimation^64,65^. In this analysis, sensor data were first smoothed by applying a low-pass filter of 50 Hz. A standardized brain^66^ (MNI-305: Montreal Neurological Institute) and the individuals’ head shape were used to co-register and construct the head models. A realistic single-shell head model was used for lead-field matrix computation. The source model consisted of dipoles located at regular 3-D grid points (1-cm separation) within the single-shell head model. Dynamic statistical parametric mapping^67^ was applied for each of the 10 stimulus conditions. The noise covariance matrix was computed using the data averaged over all conditions from − 100 to 0 ms relative to stimulus onset. To tolerate some individual differences, the source activity was temporally smoothed by a 51-ms averaging window and spatially smoothed with a Gaussian kernel (full-width half-maximum = 15 mm × 15 mm × 15 mm). Finally, the time course of source activity was down-sampled to 85.3 Hz for statistical testing.

### Time–frequency analysis

To explore the main component of time–frequency MEG responses involved in disparity-induced vergence, we applied the Fast-Fourier Transform to sensor data from individual trials with a 500-ms Hanning window. The window was shifted from − 500 to 500 ms by a 50-ms interval in the time domain and from 2 to 30 Hz by a 2-Hz interval in the frequency domain. The obtained power data were averaged across trials in each frequency and condition for each subject and log-transformed. The data were baseline-corrected by subtracting the mean log-transformed power averaged over the pre-stimulus range from − 250 to − 100 ms relative to stimulus onset.

### Brain-ocular coherence

We applied DICS^26,68^ to search and localize oscillatory brain responses correlated with the execution of vergence eye movements. Based on the results of our time–frequency analysis, we calculated the 4-Hz component of the MEG and signed vergence velocity data at each of 100–250 ms relative to stimulus onset by a 50-ms interval. A Hanning window of 500 ms was applied to the whole-brain MEG sensor data together with vergence velocity data. The MEG sensor data for the non-zero horizontal and vertical disparity conditions were coupled with the corresponding horizontal and vertical vergence velocities, respectively. The head and source models were the same as those used for the minimum-norm source reconstruction. Using the beamforming technique, we estimated the 4-Hz power and cross-spectrum between the MEG and EOG velocity data in order to calculate the strength of coherence between source activity at each location and the vergence velocity. A common spatial filter was calculated by using data from all conditions for each subject. As with the preceding analyses, coherence values were fitted with the models described below.

### Experimental design and modeling

To examine neural sensitivity to disparity and the mechanisms underlying vergence, the obtained vergence velocity, neural activity, power, and coherence were analyzed according to the following three models. In the disparity magnitude and linear disparity models, trials were categorized into 10 combinations of two disparity directions and five disparity levels (mean = 38 and min = 31 trials). The disparity magnitude model presumed that responses are linearly proportional to and correlated with the absolute value of disparity (0.00, 0.16, and 0.33 dva) in each disparity direction. The linear disparity model was identical to the disparity magnitude model except that responses are proportional to signed disparity (e.g., different signs for convergence and divergence). In the stimulus-vergence interaction model, data sets were split into trials where disparity-induced vergence occurred poorly and those where disparity-induced vergence occurred strongly (mean = 38 and min = 33 trials). This split was based on the median value of the vergence velocity averaged over the period in which significant disparity-induced vergence was found (horizontal, 150–302 ms; vertical, 232–314 ms). Relative to the disparity sign, the median vergence velocities averaged over the 29 subjects were 1.07 and 1.77 deg/s for horizontal (poor and strong vergence trials, respectively) and − 0.16 and 0.96 deg/s for vertical. The differences between poor and strong trials were not significantly greater for vertical than for horizontal (two-tailed *t* test: *t*(28) = 0.66, *p* = 0.51). The stimulus-vergence interaction model presumed that the brain-ocular coherence is correlated with the normalized strength of disparity-induced vergence (0 = poor vergence trials; 1 = strong vergence trials).

### Statistical analysis

Sensor and source data were analyzed at the group level using two-step, non-parametric cluster-based tests for a multiple-comparison correction^69^. First, *t*, *F*, or regression statistics were calculated for each sensor or voxel at a given time point. Second, clusters consisting of significant sensors or voxels (alpha level = 0.05) that were spatiotemporally adjacent to each other were subjected to permutation tests (repetition = 1000). In each step, one-tailed tests were performed for the positive tail wherever a straightforward relationship between disparity parameter and brain activity was expected. Two-tailed tests were performed where appropriate, and noted explicitly in this manuscript. For vergence data, a similar procedure was applied for the time course of velocity using regression statistics for each of the horizontal and vertical conditions. In accordance with Sassenhagen and Draschkow^70^, we reported a cluster summary for descriptive purposes.

To interpret the spatiotemporal profile of significant voxels, an obtained four-dimensional cluster was divided into two subclusters according to the *k*-means clustering algorithm. In our analysis, optimal subclusters were estimated by minimizing the sum of squared Euclidian distances with 10 repetitions.

## Data Availability

All statistical data analyzed during this study are available via the Open Science Framework (https://osf.io/n6z7h/?view_only=d8f7e93fe17343eb8272c42940cfd3be).

## References

[1] Backus BT, Banks MS, van Ee R, Crowell JA (1999). Horizontal and vertical disparity, eye position, and stereoscopic slant perception. Vision Res..

[2] Howard IP, Fang X, Allison RS, Zacher JE (2000). Effects of stimulus size and eccentricity on horizontal and vertical vergence. Exp. Brain Res..

[3] Wismeijer DA, van Ee R, Erkelens CJ (2008). Depth cues, rather than perceived depth, govern vergence. Exp. Brain Res..

[4] Backus BT, Fleet DJ, Parker AJ, Heeger DJ (2001). Human cortical activity correlates with stereoscopic depth perception. J. Neurophysiol..

[5] Brouwer GJ, van Ee R, Schwarzbach J (2005). Activation in visual cortex correlates with the awareness of stereoscopic depth. J. Neurosci..

[6] Durand JB, Peeters R, Norman JF, Todd JT, Orban GA (2009). Parietal regions processing visual 3D shape extracted from disparity. Neuroimage.

[7] Hubel DH, Wiesel TN, Yeagle EM, Lafer-Sousa R, Conway BR (2015). Binocular stereoscopy in visual areas V-2, V-3, and V-3A of the macaque monkey. Cereb. Cortex.

[8] Joly O, Vanduffel W, Orban GA (2009). The monkey ventral premotor cortex processes 3D shape from disparity. Neuroimage.

[9] Prince SJ, Cumming BG, Parker AJ (2002). Range and mechanism of encoding of horizontal disparity in macaque V1. J. Neurophysiol..

[10] Tsao DY (2003). Stereopsis activates V3A and caudal intraparietal areas in macaques and humans. Neuron.

[11] Uka T, DeAngelis GC (2004). Contribution of area MT to stereoscopic depth perception: Choice-related response modulations reflect task strategy. Neuron.

[12] Gamlin PD, Yoon K (2000). An area for vergence eye movement in primate frontal cortex. Nature.

[13] Zhang H, Gamlin PD (1998). Neurons in the posterior interposed nucleus of the cerebellum related to vergence and accommodation. I. Steady-state characteristics. J. Neurophysiol..

[14] Nitta T, Akao T, Kurkin S, Fukushima K (2008). Involvement of the cerebellar dorsal vermis in vergence eye movements in monkeys. Cereb. Cortex.

[15] Quinet J, Schultz K, May PJ, Gamlin PD (2020). Neural control of rapid binocular eye movements: Saccade-vergence burst neurons. Proc. Natl. Acad. Sci. U. S. A..

[16] Mays LE, Porter JD, Gamlin PD, Tello CA (1986). Neural control of vergence eye movements: Neurons encoding vergence velocity. J. Neurophysiol..

[17] Wojtczak-Kwasniewska M, Przekoracka-Krawczyk A, Van der Lubbe RHJ (2018). The engagement of cortical areas preceding exogenous vergence eye movements. PLoS ONE.

[18] Erkelens IM (2020). A differential role for the posterior cerebellum in the adaptive control of convergence eye movements. Brain Stimul..

[19] Busettini C, Fitzgibbon EJ, Miles FA (2001). Short-latency disparity vergence in humans. J. Neurophysiol..

[20] Rambold HA, Miles FA (2008). Human vergence eye movements to oblique disparity stimuli: Evidence for an anisotropy favoring horizontal disparities. Vision Res..

[21] Rambold HA, Sheliga BM, Miles FA (2010). Evidence from vergence eye movements that disparities defined by luminance and contrast are sensed by independent mechanisms. J. Vis..

[22] Stevenson SB, Lott LA, Yang J (1997). The influence of subject instruction on horizontal and vertical vergence tracking. Vision Res..

[23] Nikolova M, Jainta S, Blythe HI, Jones MO, Liversedge SP (2015). Vergence responses to vertical binocular disparity during lexical identification. Vision Res..

[24] Gamlin PD, Yoon K, Zhang H (1996). The role of cerebro-ponto-cerebellar pathways in the control of vergence eye movements. Eye (Lond.).

[25] Andersen LM, Jerbi K, Dalal SS (2020). Can EEG and MEG detect signals from the human cerebellum?. Neuroimage.

[26] Gross J (2001). Dynamic imaging of coherent sources: Studying neural interactions in the human brain. Proc. Natl. Acad. Sci. U. S. A..

[27] Furl N, Lohse M, Pizzorni-Ferrarese F (2017). Low-frequency oscillations employ a general coding of the spatio-temporal similarity of dynamic faces. Neuroimage.

[28] Mars RB (2008). Trial-by-trial fluctuations in the event-related electroencephalogram reflect dynamic changes in the degree of surprise. J. Neurosci..

[29] Avarvand FS (2017). Objective quality assessment of stereoscopic images with vertical disparity using EEG. J. Neural. Eng..

[30] Norcia AM, Gerhard HE, Meredith WJ (2017). Development of relative disparity sensitivity in human visual cortex. J. Neurosci..

[31] Mitsudo H, Hironaga N, Ogata K, Tobimatsu S (2019). Vertical size disparity induces enhanced neural responses in good stereo observers. Vision Res..

[32] Mitsudo H, Sakai A, Kaneko H (2013). Vertical size disparity and the correction of stereo correspondence. Perception.

[33] Preston TJ, Li S, Kourtzi Z, Welchman AE (2008). Multivoxel pattern selectivity for perceptually relevant binocular disparities in the human brain. J. Neurosci..

[34] Sprague WW, Cooper EA, Tosic I, Banks MS (2015). Stereopsis is adaptive for the natural environment. Sci. Adv..

[35] Takemura A, Murata Y, Kawano K, Miles FA (2007). Deficits in short-latency tracking eye movements after chemical lesions in monkey cortical areas MT and MST. J. Neurosci..

[36] Arnoldussen DM, Goossens J, van Den Berg AV (2015). Dissociation of retinal and headcentric disparity signals in dorsal human cortex. Front. Syst. Neurosci..

[37] Alkan Y, Biswal BB, Alvarez TL (2011). Differentiation between vergence and saccadic functional activity within the human frontal eye fields and midbrain revealed through fMRI. PLoS ONE.

[38] McCusker MC, Lew BJ, Wilson TW (2021). Three-year reliability of MEG visual and somatosensory responses. Cereb. Cortex.

[39] van Es MWJ, Marshall TR, Spaak E, Jensen O, Schoffelen JM (2020). Phasic modulation of visual representations during sustained attention. Eur. J. Neurosci..

[40] Semenova U (2021). Neuronal activity of pallidal versus cerebellar receiving thalamus in patients with cervical dystonia. Cerebellum.

[41] Giesel M (2019). Relative contributions to vergence eye movements of two binocular cues for motion-in-depth. Sci. Rep..

[42] Mitsudo H, Nakamizo S, Ono H (2005). Greater depth seen with phantom stereopsis is coded at the early stages of visual processing. Vision Res..

[43] Przekoracka-Krawczyk A, Wojtczak-Kwaśniewska M, Przekoracka K, Zeri F, Naskręcki R (2018). Sensory processing related to vergence eye movements—an event-related potential study. Opt. Appl..

[44] Balslev D, Siebner HR, Paulson OB, Kassuba T (2012). The cortical eye proprioceptive signal modulates neural activity in higher-order visual cortex as predicted by the variation in visual sensitivity. Neuroimage.

[45] van Ee R, Schor CM (2000). Unconstrained stereoscopic matching of lines. Vision Res..

[46] Mitsudo H (2007). Illusory depth induced by binocular torsional misalignment. Vision Res..

[47] Mitsudo H, Kaneko H, Nishida S (2009). Perceived depth of curved lines in the presence of cyclovergence. Vision Res..

[48] Kapoula Z (2016). Objective evaluation of vergence disorders and a research-based novel method for vergence rehabilitation. Transl. Vis. Sci. Technol..

[49] Gibaldi A, Banks MS (2019). Binocular eye movements are adapted to the natural environment. J. Neurosci..

[50] Mays LE (1984). Neural control of vergence eye movements: Convergence and divergence neurons in midbrain. J. Neurophysiol..

[51] Schor C, Wood I, Ogawa J (1984). Binocular sensory fusion is limited by spatial resolution. Vision Res..

[52] van Rijn LJ, ten Tusscher MP, de Jong I, Hendrikse F (1998). Asymmetrical vertical phorias indicating dissociated vertical deviation in subjects with normal binocular vision. Vision Res..

[53] Hironaga N, Haruhana K, Liu LC, Fenwick PBC, Ioannides AA (2004). Monitoring of eye movement and its use for artifact elimination. Int. Congr. Ser..

[54] Spekreijse H, Riemslag FCC, Carpenter RHS, Robson JG (1998). Gross potential recording methods in ophthalmology. Vision Research: A Practical Guide to Laboratory Methods.

[55] Liversedge SP, Gilchrist I, Everling S (2011). The Oxford Handbook of Eye Movements.

[56] Taulu S, Kajola M, Simola J (2004). Suppression of interference and artifacts by the signal space separation method. Brain Topogr..

[57] Taulu S, Simola J (2006). Spatiotemporal signal space separation method for rejecting nearby interference in MEG measurements. Phys. Med. Biol..

[58] Gross J (2013). Good practice for conducting and reporting MEG research. Neuroimage.

[59] Oostenveld R, Fries P, Maris E, Schoffelen JM (2011). FieldTrip: Open source software for advanced analysis of MEG, EEG, and invasive electrophysiological data. Comput. Intell. Neurosci..

[60] Bell AJ, Sejnowski TJ (1995). An information-maximization approach to blind separation and blind deconvolution. Neural Comput..

[61] Wutz A, Zazio A, Weisz N (2020). Oscillatory bursts in parietal cortex reflect dynamic attention between multiple objects and ensembles. J. Neurosci..

[62] Costers L (2021). The role of hippocampal theta oscillations in working memory impairment in multiple sclerosis. Hum. Brain Mapp..

[63] Garcés P, Lopez-Sanz D, Maestu F, Pereda E (2017). Choice of magnetometers and gradiometers after signal space separation. Sensors (Basel)..

[64] Hämäläinen MS, Ilmoniemi RJ (1994). Interpreting magnetic fields of the brain: Minimum norm estimates. Med. Biol. Eng. Comput..

[65] Hashizume A, Hironaga N, Tobimatsu S, Kakigi R (2016). Principles of magnetoencephalography. Clinical Applications of Magnetoencephalography.

[66] Collins DL, Neelin P, Peters TM, Evans AC (1994). Automatic 3D intersubject registration of MR volumetric data in standardized Talairach space. J. Comput. Assist. Tomogr..

[67] Dale AM (2000). Dynamic statistical parametric mapping: Combining fMRI and MEG for high-resolution imaging of cortical activity. Neuron.

[68] Laaksonen H, Kujala J, Salmelin R (2008). A method for spatiotemporal mapping of event-related modulation of cortical rhythmic activity. Neuroimage.

[69] Maris E, Oostenveld R (2007). Nonparametric statistical testing of EEG- and MEG-data. J. Neurosci. Methods.

[70] Sassenhagen J, Draschkow D (2019). Cluster-based permutation tests of MEG/EEG data do not establish significance of effect latency or location. Psychophysiology.

